# The Importance of Blood Pressure Observation in Surgical Prognosis

**Published:** 1920-03

**Authors:** 


### THE IMPORTANCE OF BLOOD PRESSURE OBSERVATION IN SURGICAL PROGNOSIS

Speaking before the Providence (R. I.) Medical Association, Albert H. Miller, president of the American Association of Anesthetists, drew attention to the fact that the blood pressure is the most valuable single means at the disposal of the surgical team for making a pre-operative prognosis and for judging the condition of the patient during and after operation. It may uncover arteriosclerosis, nephritis, myocarditis, aortic insufficiency, or mitral stenosis. It registers the ability to withstand hemorrhage, the depression of the anesthetic and surgical shock. Publishing his conclusions in the *Boston Medical and Surgical Journal,* 1919, Miller contends that in the present advanced state of surgical knowledge the patient has a right to expect a fairly exact pre-operative diagnosis and a very exact pre-operative prognosis. The surgeon who makes and records a prognosis before each operation and checks up his pre-operative opinion with the result will rapidly gain in skill in this important department.

Miller classifies his cases into good, fair and poor risks. Good risks—patients free from organic disease, whose surgical condition is not likely to prove fatal—are expected to recover. If a fatality occurs in this class of patients, the case should be carefully gone over to determine if the pre-operative prognosis was in error or the work of the surgical team to blame for the fatality. In fair risks—patients suffering from organic disease, but whose surgical condition is not specially serious, if no examination and no prognosis has been made, the necessity for a lame explanation of a fatality—for instance, fatal diabetic coma after appendectomy—is most deplorable. In poor risks—patients whose surgical condition is so serious or so far advanced as likely to result in fatality—recovery may be unlikely without operation, and the prospect of death should be anticipated by due warning.

In a series of one thousand consecutive operations, studied under this classification, Miller found the following results:

| | Class 1 | Class 2 | Class 3 | Total |
|---|---|---|---|---|
| Cases .................... | 734 | 179 | 87 | 1000 |
| Deaths .................... | 2 | 14 | 29 | 45 |
| Percentage ............... | .27 | 7.82 | 33.33 | 4.5 |

The deaths recorded occurred in from 24 hours to three weeks after operation. No deaths took place during or immediately following operation. Measured measure of anesthesia was used by Miller exclusively.

To determine the accuracy of Moots' rule, that if the pressure ratio (representing the relationship existing between the kinetic energy expended by the cardiac contraction in moving the blood column and the potential energy stored in the arterial walls and columns of blood which they contain) lies between 25 and 75 per cent., the case is operable, if outside these limits, probably inoperable, Miller investigated his series of one thousand cases and tabulated the results. According to Moots' rule, 3.23 per cent. of the operable cases died and 96.77 per cent. recovered. Of the inoperable cases, 23.07 per cent. died and 76.93 per cent. recovered. Some of the cases classed as inoperable underwent minor operations safely, and some of those classed as operable died after very serious operations and under circumstances which could not have been readily predicted. On an average, Miller believes that his results show the great value of Moots' rule in surgical prognosis.

McKesson's rule—that after a half-hour of sustained low blood pressure and rapid pulse, almost every patient succumbs either shortly or within three days of surgical shock and heart exhaustion—was put to a similar test. In a considerable number of cases shock (characterized by a diastolic pressure of 80 mm. or less, a pulse pressure of 20 mm. or less and a pulse rate of 120 or more) was reported by Miller to his surgeons and the operation rapidly completed. All of these patients recovered. Thirteen of the patients were in the danger zone from 25 to 70 minutes. Of these 9 died, giving a mortality rate of 69.23 per cent. These figures certainly indicate the great value of McKesson's rule for determining shock during operation.

Both rules, according to Miller's conclusions, are trustworthy and valuable aids and should be routinely employed.

